# Simulation of the Evolution of Thermal Dynamics during Selective Laser Melting and Experimental Verification Using Online Monitoring

**DOI:** 10.3390/s20164451

**Published:** 2020-08-10

**Authors:** Peiying Bian, Xiaodong Shao, Jingli Du, Fangxia Ye, Xiuping Zhang, Yaozhao Mu

**Affiliations:** 1 School of Mechano-Electronic Engineering, Xidian University, Xi’an 710071, China; shao_xiao_dong@163.com (X.S.); jldu@mail.xidian.edu.cn (J.D.); 2 Xi’an Key Laboratory on Intelligent Additive Manufacturing Technologies, Shaanxi Key Laboratory of Surface Engineering and Remanufacturing, Xi’an University, Xi’an 710065, China; yfx324@163.com (F.Y.); zhang_xp2017@126.com (X.Z.); myzo@163.com (Y.M.)

**Keywords:** selective laser melting (SLM), molten pool, moving heat source, thermal field

## Abstract

The process parameters of selective laser melting (SLM) significantly influence molten pool formation. A comprehensive understanding and analysis, from a macroscopic viewpoint, of the mechanisms underlying these technological parameters and how they affect the evolution of molten pools are presently lacking. In this study, we established a dynamic finite element simulation method for the process of molten pool formation by SLM using a dynamic moving heat source. The molten pool was generated, and the dynamic growth process of the molten pool belt and the evolution process of the thermal field of the SLM molten pool were simulated. Then, a deposition experiment that implemented a new measurement method for online monitoring involving laser supplementary light was conducted using the same process parameters as the simulation, in which high-speed images of the molten pool were acquired, including images of the pool surface and cross-section images of the deposited samples. The obtained experimental results show a good agreement with the simulation results, demonstrating the effectiveness of the proposed algorithm.

## 1. Introduction

Additive manufacturing (AM), first established over 20 years ago, is a process by which a part is built layer by layer under an applied external energy source, such as a high-energy laser beam or an electronic beam [1]. Though developments in recent decades have led to the successful achievement of certain mechanical properties and microscopic characteristics, the applications of AM remain limited. So far, only a few particular categories of AM are thought to be able to produce reliably dense parts [2,3]. Although the advantages of AM are widely recognized, many technical challenges remain to be resolved to achieve wider application in modern industry [4,5]. One of the key issues with AM is the characteristics of the molten pool induced during heat transfer in the heating and cooling cycles in the AM process because unbalanced pools readily cause thermal stresses and distortions in the additive manufactured parts [6,7], which are the major obstacles preventing AM technology from being more widely accepted [8,9].

The powder used in AM is heated through laser irradiation with a high-energy laser beam, the powder melts quickly due to rapidly reaching its melting point, forming a melting pool of a particular size corresponding to the geometric characteristics of the laser spot. Numerous physical processes occur simultaneously, such as outward heat transfer and splashing of the molten pool. Especially after the laser beam is removed, the molten pool starts to cool and solidify, during which a complex phase transition process occurs [10,11]. As the subsequent sedimentary layer is heated via heat transfer, this reheating results in a complex change in the shape of the pool. The shape of the molten pool simultaneously plays an important role in determining grain shape, dendrite growth, and thermal stress formation [12], and even affects the deformation of the part after it is finally formed.

### 1.1. Mesoscopic Simulation in Metal Powder

Many scholars have used simulation modeling to investigate the mesoscopic features occurring during selective laser melting (SLM) processes [13,14,15,16,17]. Carolin et al. [13] built a 2D lattice Boltzmann model to investigate melting and resolidification of the powder bed under the irradiation of a Gaussian beam during selective beam melting. They clearly described the process from individual powder particles to melting and refreezing. Saad et al. [14] developed a 3D mesoscopic model to simulate selective laser melting processes using the ALE3D multiphysics code. They described the melting and solidification process of the powder in addition to simulating the molten tracks under different laser powers. Chinnapat et al. [15] studied the thermal fluid flow and resulting microstructural evolution of a set of laser-scanned single tracks with different powder layer thicknesses and scanning speeds during SLM of titanium alloy powder. The increased Marangoni force and recoil pressure destabilize the melt flow in thicker powder layers. Voisin et al. [16] simulated the defects of a molten pool in the deposition of SLM powders. They confirmed the effects of defects, such as dislocation and porosity, on its tensile properties through molten pool research. The Marangoni coefficients with a fixed-point heat source were calculated by Zhang et al. [17]. They also examined the effects of convective and conductive heat flux on molten pool shape during the SLM process with a fixed-point heat source. As a whole, these investigations resulted in a massive computing project involving enormous amounts of data; as the required calculations remain infeasible at present, in-depth analyses of the pool characteristics are still lacking.

### 1.2. Thermal Flow Simulation on the Workpiece Scale

In the recent literature, most of the performed analyses have been conducted from the perspective of the workpiece, paying more attention to the macroperformance of the molten pool and the influence on future thermal performance [18,19,20,21]. Xiao et al. [18] built a 3D model describing the melting and resolidification on the scale of the SLM workpiece, considering the effects of shrinkage and natural convection driven by the surface tension and buoyancy force. The temperature distribution in the laser/powder interaction zone and the shape of the melt pool were numerically calculated using the proposed model with coupled radiation and heat transfer applicable to single vectors [19]. Yuan et al. [20] simulated the temperature evolution and the thermal behavior of the molten pool during the SLM of TiC/AlSi_10_Mg nanocomposites, which included the effects of Marangoni convection and SLM processing parameters, such as laser power and scan speed. Based on a TiAl_6_V_4_ powder system, Huang et al. [21] developed an energy conservation equation with a Gaussian energy source by considering the temperature-dependent thermal physical properties of the materials. The temperature distribution and molten pool dimensions were determined using the finite element method. In addition, Liu et al. [22] used the unique thermal behavior during the multilayer SLM manufacturing process to create the unique microstructure of as-produced parts. The molten pool size and temperature increased as the number of layers increased. Cao et al. [23] simulated the solidified track dimensions of the molten pool in nickel-based superalloys. They calculated and analyzed the influence of different process parameters (laser power and scanning speed) on the SLM formation of an Inconel 718 alloy. Mishra et al. [24] created a volumetric heat source used in numerical modeling of the SLM of Ti_6_Al_4_V powder. The same two key process parameters, laser power and scan speed, were used for single-track and multitrack SLM simulations. A group of optimal process parameters were obtained through the shape and temperature of the molten pool.

The molten pool is characterized by dynamic, rapidly changing processes. At present, point heat-transfer analysis of the pool is widely used in numerical simulation. In the studies described above, the dynamic changes and characteristics are not reflected. In addition, experimental fusion pool acquisition and verification are rarely performed. Therefore, the dynamic evolution of the molten pool and its morphology after deposition are not clearly understood. No further analyses of the characteristics of molten pools have been published.

In this study, we analyzed the evolution process of a selective laser melting pool with a dynamic heat source, and the geometry size, cooling morphology, and thermal field distribution of the pool were simulated. Based on an experiment with the same parameters, the real geometry of the molten pool formed by laser deposition was determined using a new measurement method for online monitoring involving a high-speed image sensor and laser supplementary light, and the actual morphology of the molten pool after formation was characterized using an optical microscope. The obtained molten pool data were compared with the simulation results, which demonstrated the effectiveness of the simulation method for modeling the dynamic heat source in selective laser melting.

## 2. Simulation

### 2.1. The Method

When the finite element method is used to calculate complex problems, the number of finite element grids is often very large, so data redundancy overflow often occurs during the calculation, resulting in the paralysis or suspension of the calculation. Therefore, many methods based on the finite element algorithm have been adapted to practical problems [25]. In recent years, many reports have been published on the development of the “Element Birth and Death” finite element method to solve large-scale computing problems. For the thermal coupling calculation of multilayer and multichannel selection laser melting, the grid of the front layer calculation needs to be involved in the calculation in a cycle with constant attenuation, resulting in extremely large amounts of finite element calculation data. To improve the efficiency and accuracy of the SLM thermal coupling field, the use of dynamic heat source technology is proposed, based on the birth and death technique of the finite element method.

**Figure 1:**
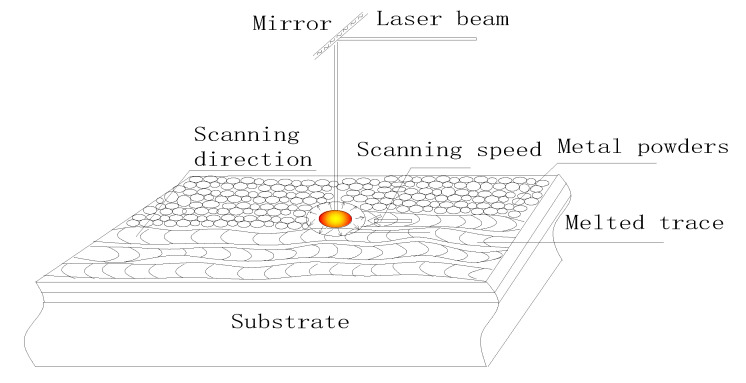
Diagram of equivalent dynamic heat source.

To achieve this, the physical implementation of dynamic heat source technology must be based on the scanning trajectory with the scanning velocity as the variable. Which is driven by the deflection of the galvanometer as the heat source; the laser beam moves along the coordinates of the scanning trajectory according to the scanning speed. Then, the laser energy is focused on the powder layer of the corresponding scanning locus coordinate point, and the material is fused to form a molten pool. This process is similar to the material point reached by the heat source being the highest temperature point of the molten pool. Therefore, the energy generated by the formed part and the energy stored in the molten pool as well as the temperature value at each location vary dynamically with the heat transfer rate. Thus, the process by which the moving laser beam acts on a point of the material can be simplified into the effect of a dynamic heat source, as shown in Figure 1.

**Figure 2:**
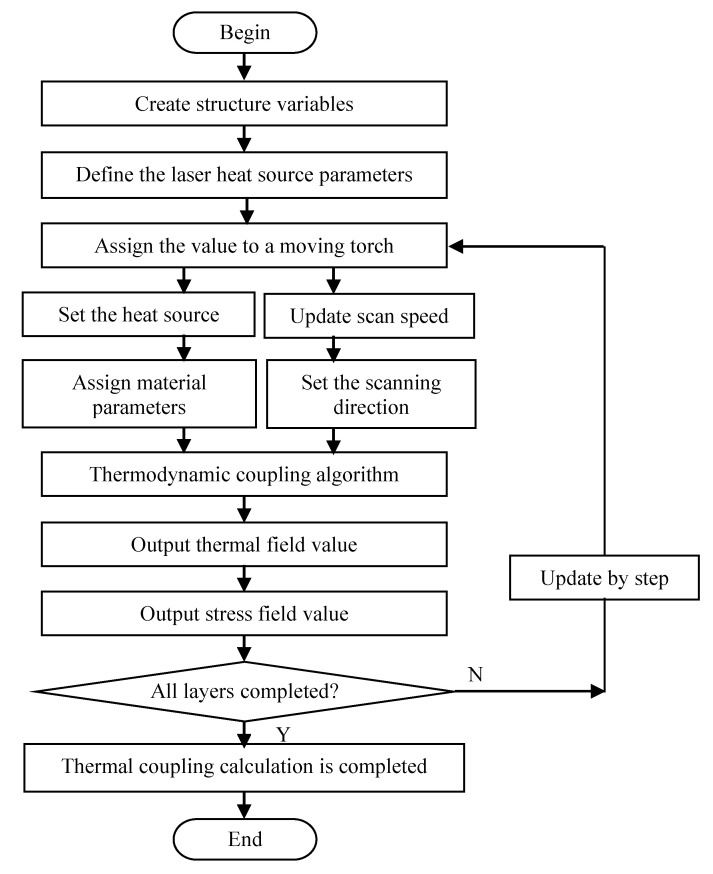
The flow of dynamic heat source algorithm.

The algorithm implemented for the dynamic heat source technology during SLM is shown in Figure 2. First, a geometric model of heat source is constructed followed by the setting of heat source parameters (process parameters). Then, the laser device is started and the heat source moves along the scanning trajectory at the scanning speed. The thermal coupling effect occurs for different molten pools. For the simulation, the heat source was established on the basis of geometry parameters and the thermal field value can be defined according to structure variables, including temperature, speed, and geometric parameters. A finite element value was assigned for moving along the scanning trajectory coordinates according to the scanning speed welding torch tools. The moving heat source parameter value was used to update the calculation for laser deflection simulations. Finally, the thermal field parameters, such as trajectory position and temperature of the thermal traversal of the welding gun, are constantly updated, that is, the temperature value of each coordinate point on the scanning trajectory is updated according to the heat transfer rate with the scanning velocity to determine the effect of the dynamic heat source from the perspective of relative motion.

### 2.2. Formula

#### 2.2.1. The Definition of the Laser Source

In this paper, the process parameters corresponding to selective laser melting are taken as the main input conditions for the simulation, which refer to the laser source. According to the characteristics of laser emission, the laser source model is the Gaussian heat source [26]:

(1) $$\;q=q_{0}exp\left(-\frac{2r^{2}}{\theta^{2}}\right)$$

where *q* is the heat flux (J/(m^2^∙s)), *r* is the radiating distance from the center, *θ* is the radius of the beam (mm), and $q_{0}$ is the maximum heat flux at the center of the beam, *r* = 0, defined as:

(2) $$q_{0}=\frac{2P\xi }{\pi \theta^{2}}$$

Hence,

(3) $$q_{\left(r\right)}=\frac{2\xi P}{\pi \theta^{2}}exp\left(-\frac{2r^{2}}{\theta^{2}}\right)$$

where *P* is the laser power (in W) and *ξ* is the laser absorptivity of metal materials (that of a 316 L stainless steel is generally taken to be 0.7).

**Figure 3:**
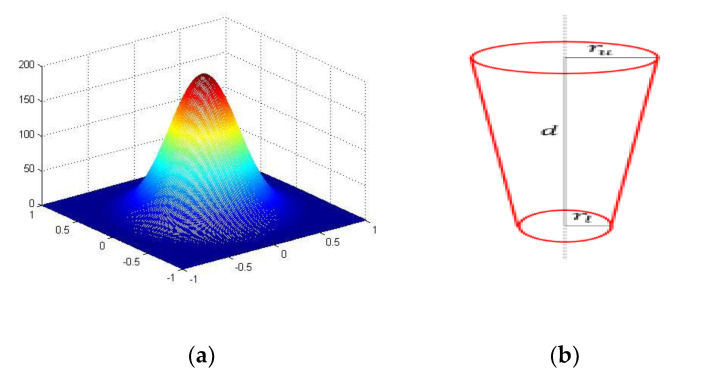
Laser heat source model: (**a**) Gaussian heat source; (**b**) geometric model of heat source.

**Figure 4:**
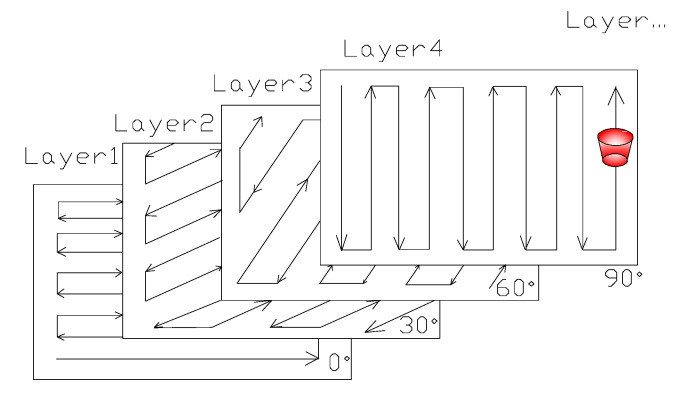
The laser scanning path of layer by layer.

The heat flux value of the laser heat source can be determined according to parameters such as laser power and spot size. The heat flux model can be expressed as shown in Figure 3a. The heat flux at the center is the highest where the heat transfer energy to the surrounding area is the highest, then gradually decreases outward in all directions. The geometric model of the heat source is shown in Figure 3b, which is an inverted cone. The geometry of the heat source can be defined by three geometric parameters: Surface radius R1, bottom radius R2, and heat source height D. The inverted cone heat source defined by the three geometric parameters moves along the scanning path according to the scanning speed, and the corresponding heat source moves along the scanning path, as shown in Figure 4. For example, under the strip-type scanning path in Figure 4, the heat source model moves layer by layer, with a deflection of 30° from 0 to 180° (0°, 30°, 60°, 90°, 120°, 0 °, 30°, etc.)

#### 2.2.2. The Model of Cycle Heat Transfer

In nature, as long as there is a transmission medium and a temperature difference between different parts of an object, then heat will be transferred from areas of high to low temperature. When the metal powder is irradiated by a laser beam, it generates heat that is stored and then transmitted. According to the basic theory of heat transfer, the three main methods of natural heat transfer are conduction, convection, and radiation [27].

1. Conduction

Generally, conduction is the transfer of heat within an object through the collision of molecules, atoms, or electrons in the material. According to the general definition of heat flux, the amount of heat passing through a substance per unit area of time can be calculated using Equations (2)–(4). Considering the X direction as an example, the heat flux transferred during the conduction process is:

(4) $$\begin{array}{cc} q=\frac{Q}{A\cdot t} & \left(Q=k_{x}A\frac{\partial T}{\partial x}\approx k_{x}A\frac{∆T}{d_{r}}\right) \end{array}$$

where *Q* is the heat, *K_x_* is the thermal conductivity in the X direction (W/(m·K)), *A* is the area through which heat flows in the X direction, *t* is the generation time corresponding to the heat transfer temperature difference, *T* is the surface temperature of the object (K), Δ*T* is the difference in temperature between a hot object and a cold object, and *d_r_* is the material thickness of the current heat transfer layer (mm). Hence, the temperature gradient is approximately expressed as:

(5) $$∆T=\frac{q_{\left(r\right)}}{k_{x}}d_{r}$$

The heat flux at each node of the model can be calculated according to the given laser power and other parameters through Equations (2) and (3); thus, the temperature gradient between each material point can be obtained from Equations (2)–(5).

2. Convection

Convection is the transfer of heat by an object to another object through a fluid, or to an objective flow via another medium. The heat flux corresponding to the convection can generally be expressed as:

(6) $$q=hA_{p}\left(T-T_{\infty }\right)$$

where *h* is the heat conduction coefficient and $T_{\infty }$ is the temperature of the surrounding convective environment.

3. Radiation

Radiant heat transfer is the process of heat transfer from a formed object to its surroundings. The radiant heat flux is determined by the following relation:

(7) $$q=\chi \psi A_{p}\left(T-T_{\infty }\right)$$

where χ is the radiation Steffen–Boltzmann constant and ψ is surface emissivity.

4. Energy produced in solids

When the laser power is turned on, heat is generated in the area of the powder where the laser beam is focused. The heat energy generated is the heat flux intensity multiplied by the volume:

(8) $$E_{g}=\dot{q}V$$

where $\;\dot{q}$ is the heat flux, which is equal to the heat rate generated per unit volume in unit time close to the heat flux, and v is the volume of the heated object.

5. Energy stored in solids

When the metal powder produces heat and as the energy continues to increase with temperature, the proportion of heat stored in the solid is related to the material’s relative heat coefficient:

(9) $$E_{s}=\rho cV\frac{\partial T}{\partial t}$$

where *ρ* is the density of the material (kg/m^3^) and *c* is the specific heat of the material (J/(kg·K)). Thus, the heat transfer equation per unit volume is obtained:

(10) $$\rho c_{p}\frac{\partial T}{\partial t}=\nabla \left(-k∆T\right)$$

where *c_p_* is the specific heat corresponding to the temperature of the current material point. Considering the cyclic heat transfer of the current layer by the preformed layer, the composite cyclic heat transfer equation is obtained:

(11) $$\rho c_{p}\frac{\partial T}{\partial t}=\overset{n}{\underset{i=1}{\sum }}\nabla_{i}\left(-k∆T_{i}\right)$$

where *i* is the number of layers of heat transfer, which is determined by the error of temperature, Δ*T_i_* < *ε*, according to the heat-affected area.

The porosity of the powder can be calculated as:

(12) $$\phi =\frac{\rho_{bulk}-\rho_{power}}{\rho_{bulk}}$$

Hence,

(13) $$\rho_{power}=\left(1-\phi \right)\rho_{bulk}\;K_{power}=\left(1-\phi \right)K_{bulk}$$

According to the literature [28], the heat balance equation satisfies the following classical three-dimensional heat conduction equation:

(14) $$\frac{\partial }{\partial x}\left(k_{x}\frac{\partial T}{\partial x}\right)+\frac{\partial }{\partial y}\left(k_{y}\frac{\partial T}{\partial y}\right)+\frac{\partial }{\partial z}\left(k_{z}\frac{\partial T}{\partial z}\right)+\dot{q}=\rho c\frac{\partial T}{\partial t}$$

Equations (2)–(14) are common differential equations for heat conduction in orthotropic materials. If the heat conductivities in the three directions X, Y, and Z are the same, the isotropic hypothesis can be applied to the material. At this point, *k_X_* = *k_Y_* = *k_Z_* = *k*; then, Equations (2)–(14) can be written as:

(15) $$\frac{\partial^{2}T}{\partial x^{2}}+\frac{\partial^{2}T}{\partial y^{2}}+\frac{\partial^{2}T}{\partial z^{2}}+\frac{\dot{q}}{k}=\frac{1}{\alpha }\frac{\partial T}{\partial t}$$

Enthalpy H can be used to calculate the heat absorption and cooling process with each time step. It is a function of density *ρ* and specific heat *C*, commonly expressed as:

(16) H=∫ρc(T)dT

6. The thermal boundary conditions

In SLM forming, the initial temperature setting, initial characteristic parameter setting of the material, initial displacement conditions, and heat transfer of the material at the boundary to the surrounding environment during deposition constitute the boundary of the heat transfer calculation model:

(17) $$T\left(x,y,z,t\right)=T_{0}$$

The boundary conditions of the simulation model are determined by considering the natural convection and surrounding radiation. The heat flux at the boundary is equal to the laser heat flux at the boundary element:

(18) $$-k{\left(\frac{\partial T}{\partial n}\right)}_{w}=q_{\left(r\right)w}$$

where *n* is the normal direction of model surface and w is in the boundary of the model. In SLM forming, the heat transfer between the sample and the surrounding environment conforms to the third boundary condition. In other words, the heat flux at the boundary is equal to the convective heat flux plus the radiant heat flux at the boundary of the sample to the surrounding environment.

(19) $$-k{\left(\frac{\partial T}{\partial n}\right)}_{w}=h\left(T_{w}-T_{f}\right)+\chi \psi \left(T_{w}^{4}-T_{f}^{4}\right)$$

### 2.3. Results

#### 2.3.1. Molten Pool Shape

**Figure 5:**
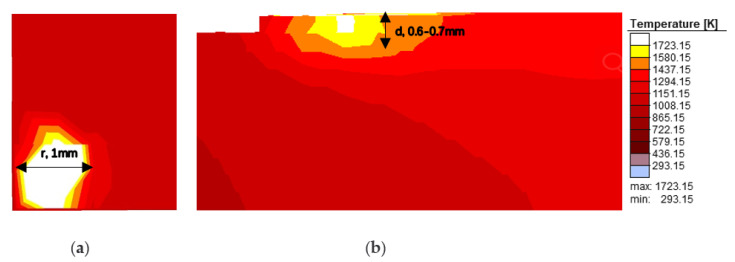
Morphology of molten pool under thermal action of the laser heat source: (**a**) Top view; (**b**) Section view.

**Figure 6:**
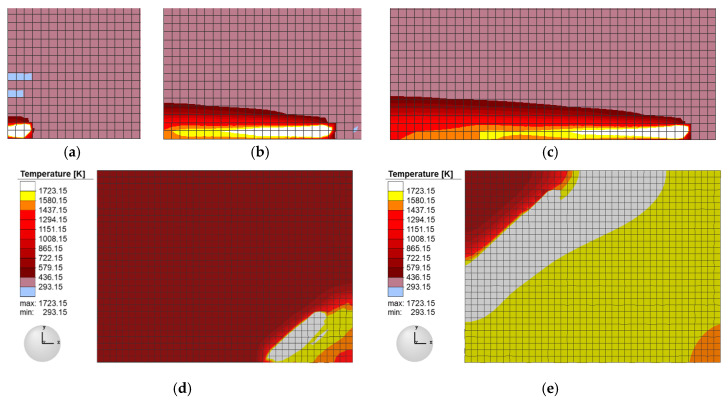
The laser heat source grows along the scanning trajectory at (**a**) 0.2 mm; (**b**) 2 mm; (**c**) 3 mm; (**d**) multitrack; and (**e**) multilayer.

The 3D model for formation was imported according to the construction of the aforementioned SLM dynamic simulation platform. The simulation model is prismatic, with dimensions of about 5 × 3 × 2 mm^3^ (length, width, height). The process parameters were power 200 W (laser spot was 0.1 mm), scanning speed 500 mm/s, overlap of about 10%, thickness of 50 μm, and striped scanning strategy. The simulation was performed on an 8 core PC with 3.60 GHz CPU, and 32 GB memory. The dynamic heat source was loaded and assigned to the welding gun, then the Gaussian heat source model was used to simulate the heat transfer from the laser beam to the forming area. Due to the high intensity and accumulation of laser energy, the temperature of the current point of the material where the beam was focused quickly reached over 2000 K, and a molten pool with certain length, width, and height formed immediately. The simulated molten pool had an inverted cone shape, as shown in Figure 5. The corresponding top view and section views of the molten pool are shown in Figure 5a,b, respectively. Its geometric shape conforms to the design goal in Figure 3b. As the scanning speed is fast and the heat conduction is relatively slow, the heat transfer in the current pool quickly connects to the next pool. Therefore, the size of the molten pool expands rapidly, as shown in Figure 6, forming the molten pool belt.

The growth process of the molten pool is shown in Figure 6a–c, which corresponds to snapshots of the moving laser beam captured along the scanning track during the moving process at 0.2, 2, and 3 mm with a speed of 500 mm/s. In addition, a new layer of powder is laid on top of the previous layer of finished powder according to the scanning path and speed. The new track is further melted along the path specified by the moving laser beam, and the previous track is heated by heat transfer from the new track, as shown in Figure 6d–e. In the same way, the new molten layer conducts heat to the sediments in front of it, allowing heat to accumulate. The dynamic, thermally coupled melting thermal field and thermal stress field of the SLM process affect formation under the thermal action of this dynamic molten pool.

#### 2.3.2. Thermal Field Distribution

**Figure 7:**
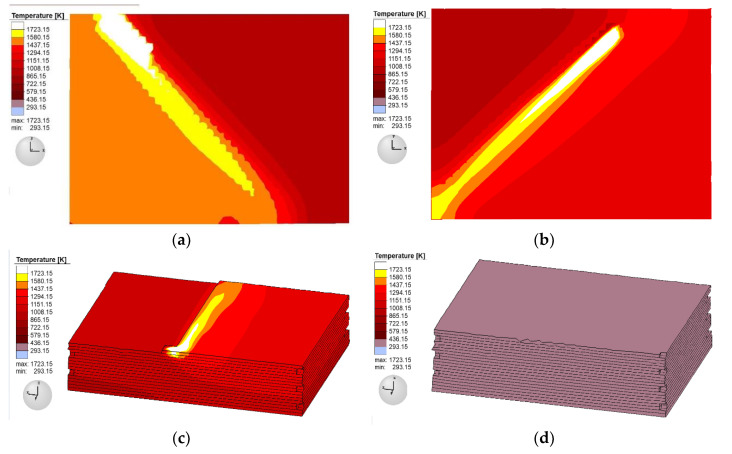
Thermal field distribution at different positions in the simulation at (**a**) 10 layers; (**b**) 20 layers; (**c**) 38 layers; and (**d**) 40 layers.

Figure 7a–d shows the cloud map results of four different forming layers at different times in the thermal field of the simulation model corresponding to SLM and the deposition process. The thermal distribution position with 10, 20, 38, and 40 layers at 12, 25, 48, and 200 s are respectively shown in Figure 7a–d. As shown in the figure, when the laser heat source irradiated the material point, the temperature rose rapidly to 2100 K, exceeding the melting temperature of 316 L stainless steel (1672 K). The metal powder immediately melted and formed a melting pool. Then, as the laser light source moved to the next position according to the scanning speed, the molten pool continued to form a molten pool belt. When the temperature dropped steadily to about 1000 K, the new layer was then heated up again. As such, the temperature gradient between different material points on the sample formed after the heat transfer of the rapidly moving heat source.

Figure 7 also shows that the molten pool zone had an obvious influence on the surrounding heat affected area, and the horizontal direction expanded to the surrounding melting channel, such that the melting channel in the front channel was partially remelted by heat transfer, and the unheated powder was preheated in advance. There were also four to five layers of a heat-affected zone down the pool along the section direction, thus heating and remelting the previously formed layer. This indicates that the thermal field is involved in the cumulative calculation in the dynamic heat source and cyclic temperature accumulation model, and that the aforementioned dynamic heat source and cyclic heat transfer model is more in line with the actual thermal field distribution. After all the forming layers were scanned, the whole sample radiated heat to the surrounding area through convection and soon cooled to room temperature.

## 3. Online Monitor

### 3.1. The Method of Monitor

**Figure 8:**
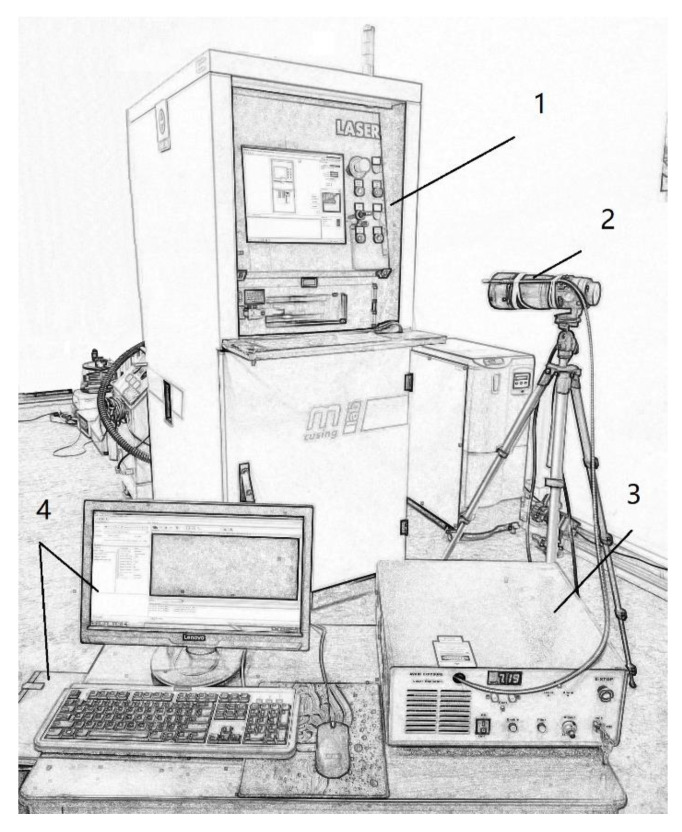
The method used to measure the dynamic molten pool. 1-Selective laser melting (SLM) machine; 2-camera; 3-laser generator; 4-computer.

The dynamic molten pool was first measured with a high-speed camera during the process of deposition. The morphology of the molten pool was photographed using light supplemented from a laser. The main specifications of the high-speed camera are resolution of 1280 × 1024 pixels, frame frequency of 500–33, 390 fps, dynamic range of 120 dB, and minimum exposure time of 1 μs; the picture element was 14 × 14 μm, capture card was a PCI e2.0 × 4, and the CMOS sensor achieved high sensitivity even at 2500. For the laser supplementary light, the laser generator power was 100 W, and laser spotsize was 0.4 mm with a fiber length of 2 m. The measurement method is shown in Figure 8. Next, the static shape of molten pool was observed using an HIROX KH-1300 3D optical microscope (Hirox, Tokyo, Japan). The surface morphology of the cooled molten pool was captured. Then, the cross-section morphology of the molten pool was captured on an optical microscope prepared by polishing the formed specimen. The formation of the three-dimensional angle of the molten pool morphology was used to analyze characteristics.

### 3.2. Results

#### 3.2.1. The Dynamic Molten Pool Shape

**Figure 9:**
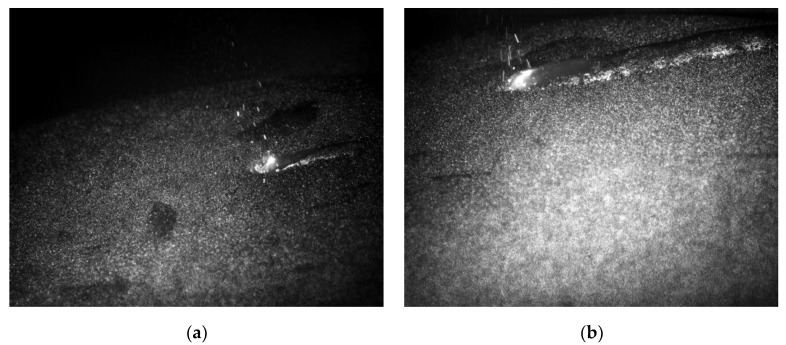
Snapshot of the online molten pool under SLM formation: (**a**) Molten pool; (**b**) Molten pool belt.

The SLM formation experiment was conducted using the same process parameters as in the simulation. A high-speed image sensor and a laser fill-in light for online monitoring were used, as shown in Figure 8. The laser fiber was loaded onto the camera to enable high-speed photography, which led to the successful capture of the SLM fast-exposure image of the molten pool corresponding to the laser processing parameters detailed in this paper. The laser penetrates the surface powder and moves along the scanning path and a banding pool is then formed, as shown in Figure 9a,b. The banding pool is similar to the strip pool belt simulated by the dynamic heat source in the simulation, as shown in Figure 6 and Figure 7. The current exposure point was nearly an inverted cone due to the rapid scanning speed, so the front hot pool had no time to solidify and mixed with the current pool to form the tail of the pool, forming the strip pool.

#### 3.2.2. The Static Molten Pool Shape Geometry

**Figure 10:**
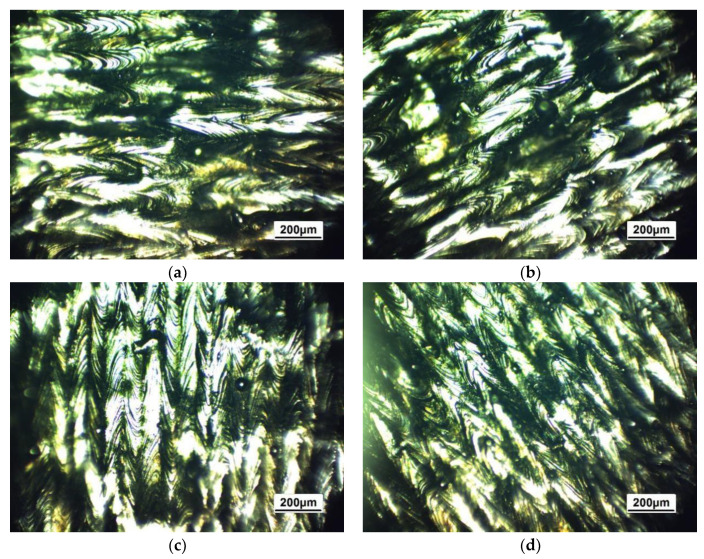
Morphology of the surface molten pool after different angles of SLM formation: (**a**) 0°; (**b**) 30°; (**c**) 90°; and (**d**) 120°.

After the sample was cooled, the surface morphology of the solidified molten pool was clearly observed under an optical microscope without remelting. The cooling marks produced a series of roundabout lines due to the Marangoni convective effect in the molten pool when the light spot cooled down, as shown in Figure 10. In addition, the different forming angles are described in detail in Figure 10a–d. The sample surface was not particularly uniform due to the concentration of powder. Therefore, a rough surface formed along the core and intermediate lap joints of the molten pool.

**Figure 11:**
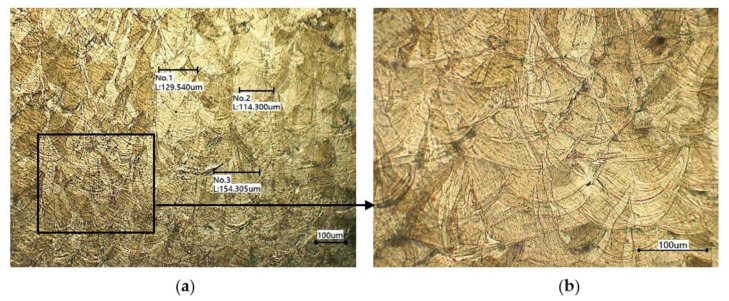
Morphology of the cross-section molten pool after SLM formation: (**a**) Cross-section; (**b**) Partial enlarged detail.

The molten pool morphology in the direction of YOZ section of the sample is shown in Figure 11. In the adjacent weld channels of the same layer of the molten pool in the macroscopic observation area, the effect of the laser lap rate, cross-solidification with each other, and lap marks are clearly visible. The bottom circle of the upper molten pool extends to the surface of the lower solidified molten pool, superimposing layer upon layer. Therefore, the overall optical microscope image showed that after solidification, the layers still had a fish-scale distribution, which accumulated after the solidification of the strip pool belt layer by layer. The pool channel that was actually formed also corresponds with predictions from the simulation results.

The combined results of the online monitoring images and the surface cross-section pictures of the molten pool are interesting. Under laser irradiation, the material points are heated to form a molten pool and then a molten pool belt in a short time. After cooling, according to the analysis of the molten pool, the silted lines were formed after evaporation of the surface, the fish-scales were formed due to the shape of the molten pool with overlapping cross-sectional layers. The dynamic evolution effect on the molten pool verifies that the simulation effect is consistent with the actual forming process.

## 4. Discussion

**Figure 12:**
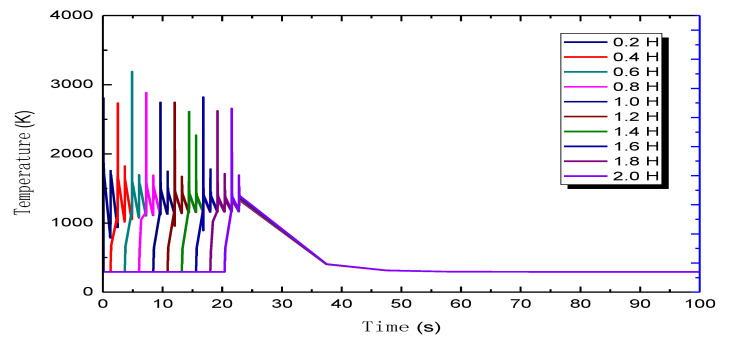
The temperature cycle curve of the numerical simulation at a mid-point in the sample. H is the height of SLM sample in the simulation, the curve is actually the time in X-axis corresponding to the temperature in Y-axis.

In this paper, we proposed a dynamic thermal coupling simulation method for SLM formation driven by process parameters. The dynamic thermal coupling simulation data were analyzed using the process parameters of a cylindrical 316 L stainless steel sample as an example. The evolution of the molten pool is analyzed in detail below. Figure 12 shows the temperature change process of several representative tracking points at the same *X* and *Y* coordinates of different layers in the center of the simulation sample. As can be seen from the comparison in Figure 12, the initial temperature of the first layer was that of the initial room temperature. When the laser heat source moved to the tracking point, the temperature rose rapidly to nearly 2200 K, which exceeds the melting temperature of 316 L stainless steel (1672 K). Then, as the laser light source moved to the next position and heat was transferred around the hot part of the molten pool, the temperature rapidly dropped to below 1000 K, corresponding to a very large temperature gradient. When a new layer of powder was laid on the top of the material point and the laser beam scanned to the new layer of the point, the new molten pool continued to form and rapidly expanded to the adjacent solidified layer. Due to the heat transfer of the new layer, the temperature of the front layer increased again. This diffusion led to an increase in the geometry of the pool, known as remelting. After such cyclic accumulation, the diffusion depth of the molten pool was about four layers, i.e., 200 μm, as shown in Figure 12 (remelting temperature > 1672 K), which is the same as the geometric size of the molten pool measured in a previously reported experiment [29]. However, a special state appeared as the number of new layers increased, the remelting temperature decreased gradually, and the increase or decrease in temperature was less than that of the previous stage. The thermal influence of the newly formed layer on the formed layer in the early stage circulated along with the subsequent formation process, resulting in the remelting phenomena of material heating–cooling–reheating, which is referred to as cyclic heat transfer in this paper. In addition, subsequent heating by circulating heat may produce a micro-annealing effect on the preformed underlying material, which is an interesting topic for further future research. At about 50 s into the simulation of the shape process, the 40 layers of all the samples had formed, and the samples entered the pure cooling stage, whereby the alternating temperature cycle stopped. Finally, with continuous heat transfer to the surrounding area, the temperature of the whole simulation sample decreased to room temperature. The attenuation cycles for the other observation points were similar. The number of circulating layers, or diffusion depth, depends on the number of forming layers and laser energy intensity at the monitored material points.

**Figure 13:**
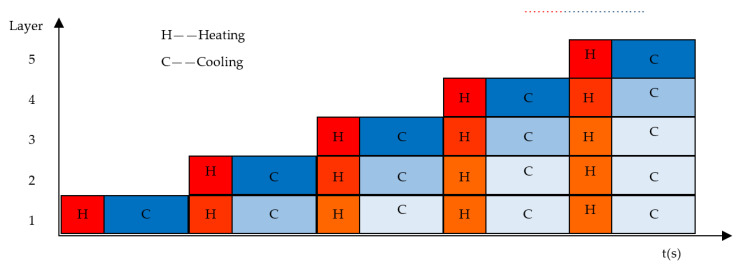
The time component diagram of the part of layer and its corresponding thermal superposition effect diagram from the simulation. The red color is the heating time of SLM sample in simulation, the blue color is cooling time. In addition, the darker the color, the higher the temperature gradient.

As shown by the evolution process of the temperature cycle curve, the temperature curve of the front layer is a multiperiod falling curve with cyclic oscillation in the evolution process from the first layer to the following layer. The curve of each layer rises and falls according to the duration of laser scanning throughout the deposition process until cooling. Finally, all layers are cooled to room temperature. The heating and cooling diagrams for each layer of the sample during the deposition process at the observation point of the five layers are shown in Figure 13. The red column expresses heating and warming during the laser exposure of the current layer and the influence on the cycling heat of the previous layers. The blue column represents the cooling of the current layer during cooling and further cooling of the thermal effects during the gradual cooling of the preceding layers. This generates the cyclic heat effect during the laser scanning process of 40 layers. After the simulation is completed, there is a certain pure cooling time where the workpiece cools to room temperature. Under the action of this high-energy rapid scanning heat source, the material instantaneously formed a higher temperature gradient △*T* with about a 10^−6^ k/s order of magnitude [30], which inevitably led to the formation of greater thermal stress inside the formed part [31].

Online monitoring of an SLM rapid scanning process was one of the central challenges [32]. In this paper, a CMOS high-speed image sensor and laser supplementary light were used to record the melting pool that completes the high scanning speed of SLM forming process under the acquisition of rapid exposure. There are two main methods for online monitoring discussed in this article. The first is the adoption of a high-speed camera, for which an acquisition frequency of a single image can reach about 1 μs; so a movement at a speed of 500 mm/s can be tracked. The second method, is to use a laser with a high instantaneity, which is a high concentration of light energy in time; therefore, the laser pulse in the order of ns (even ps or fs) is easy to achieve. With the development of laser pulse compression or ultra-short pulse technology, laser pulses are getting increasingly narrow. Such laser pulses can provide insight into many transient processes (also known as ultra-short processes), including rapid changes in the SLM molten pool.

## 5. Conclusions

In this paper, we designed a dynamic heat source and developed a circulating heat transfer model for the SLM formation process, and SLM was dynamically simulated using the finite element software. In addition, a high-speed image sensor was used to monitor the geometry of the molten pool online through an experiment based on the same process parameters. The overlapping relationship between the pool and the adjacent channel was determined by identifying the cross-section of the pool after deposition. The validity of the simulation algorithm was demonstrated by the experiments. Our main conclusions are as follows:

- A SLM dynamic heat source model similar to the working principle of a real laser heat source was established, and the motion process of molten pool was realized according to the scanning trajectory at the scanning speed.
- The heat transfer process of the SLM heat source was analyzed and demonstrated. Then, the equivalent calculation of the energy of the heat source was carried out. A simulation model of isotropic assumption was constructed for the heat transfer. Altogether, these established the foundation for the algorithm for heat transfer in the simulation process.
- According to the SLM dynamic heat transfer process simulation analysis, we defined the formation of the molten pool under the action of heat source, the growth of the molten pool, and the process of the molten pool moving according to the scanning trajectory.
- The heat transfer characteristics of the molten pool were simulated, showing that the cyclic heat transfer extended to the preformed layer. Its amplitude gradually attenuated according to the distance between the formed layer and the light source.
- A new camera with high-speed CMOS image sensor and a laser fill-in light used online monitoring of the SLM deposition processing in the experiment. The shape of the dynamics of the molten pool is captured successfully after setting the power of filling laser to a scanning speed which is suitable to monitoring the SLM formation process. Therefore, the new high-speed image sensor acquisition experiment verifies the effectiveness of the simulation algorithm.
